# Normal Ethanol Sensitivity and Rapid Tolerance Require the G Protein Receptor Kinase 2 in Ellipsoid Body Neurons in *Drosophila*

**DOI:** 10.1111/acer.14396

**Published:** 2020-07-09

**Authors:** Yuan Yuan Kang, Yusuke Wachi, Elizabeth Engdorf, Emiliano Fumagalli, Youyou Wang, Jennifer Myers, Shebna Massey, Antony Greiss, Shiyu Xu, Gregg Roman

**Affiliations:** ^1^ University of Houston Downtown Houston Texas; ^2^ University of Mississippi University Mississippi; ^3^ University of Houston Houston Texas

**Keywords:** G Protein, Coupled Receptor Kinase, Ethanol Sensitivity, Rapid Tolerance, Drosophila, Ellipsoid Body

## Abstract

**Background:**

G protein signaling pathways are key neuromodulatory mechanisms for behaviors and neurological functions that affect the impact of ethanol (EtOH) on locomotion, arousal, and synaptic plasticity. Here, we report a novel role for the *Drosophila* G protein–coupled receptor kinase 2 (GPRK2) as a member of the GRK4/5/6 subfamily in modulating EtOH‐induced behaviors.

**Methods:**

We studied the requirement of *Drosophila Gprk2* for naïve sensitivity to EtOH sedation and ability of the fly to develop rapid tolerance after a single exposure to EtOH, using the loss of righting reflex (LORR) and fly group activity monitor (FlyGrAM) assays.

**Results:**

Loss‐of‐function *Gprk2* mutants demonstrate an increase in alcohol‐induced hyperactivity, reduced sensitivity to the sedative effects of EtOH, and diminished rapid tolerance after a single intoxicating exposure. The requirement for *Gprk2* in EtOH sedation and rapid tolerance maps to ellipsoid body neurons within the *Drosophila* brain, suggesting that wild‐type *Gprk2* is required for modulation of locomotion and alertness. However, even though *Gprk2* loss of function leads to decreased and fragmented sleep, this change in the sleep state does not depend on *Gprk2* expression in the ellipsoid body.

**Conclusion:**

Our work on GPRK2 has established a role for this GRK4/5/6 subfamily member in EtOH sensitivity and rapid tolerance.

## Graphical abstract

Neuroplasticity is linked to alcohol use disorders (AUDs). One key mechanism of synaptic plasticity involves the regulation of G protein coupled receptors and downstream pathways. *Drosophila* G protein coupled receptor kinase 2 (GPRK2) is required for normal ethanol sensitivity and developing rapid tolerance after a single exposure to ethanol. This requirement is mapped to the ellipsoid body (EB) neurons, a brain region which has been linked to locomotion, sleep and arousal.

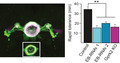

Alcohol use disorders (AUDs) are multifactorial conditions with strong genetic components and complex behavioral outputs that include alcohol abuse and addiction (Edenberg and Foroud, 2014). A low naive sensitivity to this drug, especially to its negative effects, is a risk factor for alcoholism (Morean and Corbin, 2010; Ray et al., 2010; Schuckit, 1994). Identifying the genes and signaling pathways that govern alcohol‐induced responses can help drive our understanding of the genetic susceptibility to AUDs. Many of those genes have conserved functions in *Drosophila* and vertebrates, including those encoding for alcohol dehydrogenase (*Adh*), BK channels (*slo*), cyclic AMP signaling pathway genes (*DCO*, *rutabaga*), and the genes for a postsynaptic scaffold protein (*homer*) and innate immune system (*Toll*.* cactus*.* NF‐kappaB*) (Grotewiel and Bettinger, 2015; Troutwine et al., 2016). Some genes, such as *rutabaga*, *slowpoke*. and *homer*, function in more than one alcohol‐induced behavior (Ghezzi et al., 2013; Moore et al., 1998; Urizar et al., 2007; Xu et al., 2012).

Cumulative evidence from several model systems, including mice and *Drosophila*. has suggested that the cAMP‐dependent signal transduction pathway involving protein kinase (PKA) plays an important role in the modulation of alcohol‐induced responses (Moore et al., 1998; Peng et al., 2009; Yang et al., 2003). In mice, reduced cAMP‐PKA activity leads to increased sensitivity to the sedating effect of alcohol, whereas the upregulation of the pathway has the opposite effect (Maas et al., 2005; Wand et al., 2001). In flies, genetic reduction of several genes in the cAMP‐PKA pathway: *amnesiac* (encoding a neuropeptide that induces cAMP production).* rutabaga* (encoding a calcium sensitive isoform of adenylyl cyclase), and *DCO* (encoding a subunit of PKA) all showed increased sensitivity to ethanol (EtOH) (Moore et al., 1998; Park et al., 2000; Rodan et al., 2002). Moreover, an increase in the Toll immune signaling pathway correlates with lower sensitivity to alcohol (Troutwine et al., 2016), consistent with a mammalian study that linked the Toll‐like pathway to alcohol consumption (Robinson et al., 2014). In this paper, we explore the function of the G protein–coupled receptor kinase 2 (GPRK2) in mediating alcohol‐related behaviors based on previous work that associates the activity of this kinase with the cAMP and Toll pathways (Cheng et al., 2012; Schneider and Spradling, 1997; Valanne et al., 2010).

G protein–coupled receptor kinases (GRKs) are a kinase family that regulates G protein–coupled receptors (GPCRs) through ligand binding–induced phosphorylation; this phosphorylation event leads to β‐arrestin binding and receptor desensitization (Moore et al., 2007). β‐arrestin is a nonvisual arrestin with a single β‐arrestin in *Drosophila*, encoded by *kurtz*, and 2 β‐arrestins in mice (Lefkowitz and Shenoy, 2005; Roman et al., 2000). The nonvisual β‐arrestin 2 knockout mice were less sensitive to alcohol sedation and showed a marked increase in alcohol consumption, consistent with the inverse relationship between alcohol sensitivity and alcohol abuse (Li et al., 2013; Schuckit, 1994). Some GRKs can also internalize GPCRs independent of β‐arrestin or regulate signaling in a phosphorylation‐independent manner (Evron et al., 2012).

There are 7 GRKs in mammalian species; 2 members in *C*.* elegans*, Ce‐GRK‐1 and Ce‐GRK‐2; and 2 in *Drosophila*, GPRK1 and GPRK2 (Cassill et al., 1991; Cheng et al., 2010; Fukuto et al., 2004; Homan and Tesmer, 2014). In *Drosophila*, *Gprk1* is expressed ubiquitously, whereas *Gprk2* expression is mostly restricted to neurons (Cheng et al., 2010). GPRK2 is most closely homologous to the mammalian GRK4/5/6 subfamily and is important for development, rhythmic olfactory response, and mediating immune responses to bacterial infection (Cheng et al., 2010; Schneider and Spradling, 1997; Tanoue et al., 2008; Valanne et al., 2010). During egg morphogenesis and wing development, reduced *Gprk2* function led to a low cAMP activity (Cheng et al., 2010, 2012; Schneider and Spradling, 1997). The knockdown of *Gprk2* in the *Drosophila* fat body resulted in a reduction of the Toll immune pathway (Valanne et al., 2010). Based on the role of β‐arrestin 2 in alcohol‐induced behaviors, the ability of *Gprk2* to affect cAMP levels, and the importance of PKA signaling in alcohol‐induced behaviors, we hypothesized that mutations in *Gprk2* should alter sensitivity to the sedative effects of EtOH, possibly leading to higher sensitivity in flies.

## Materials and Methods

### *Drosophila* Stocks

Fly stocks and crosses were cultured on standard cornmeal medium and maintained in a 25°C incubator with a 12‐h/12‐h light:dark cycle. All transgenic fly strains were backcrossed to a *Canton‐S* background for 6 generations. All flies examined for behavior contained a wild‐type *w^+^* X‐chromosome. The *gprk2^KO^* and *gprk2^del1^* mutants (Cheng et al., 2010; Tanoue et al., 2008) were gifts from David Hipfner (McGill University, Montreal, CA); *UAS‐Gprk2* (Cheng et al., 2010; Tanoue et al., 2008) was gifted by Paul Hardin (Texas A&M, College Station, TX); 2 *UAS‐Gprk2RNAi* lines 101463 (Dietzl et al., 2007) and *GL00233* (Perkins et al., 2015) were obtained from Bloomington and Vienna stock centers; Gal4 drivers *c819* (Renn et al., 1999) and *ruslanGal4* (Krashes and Waddell, 2008) were described previously; and *5*.*30* and *4*.*67* were generous gifts from Fred Wolf (University of California Merced, CA) (Kong et al., 2010a).

### Alcohol Loss of Righting Assay and Rapid Tolerance

The loss of righting reflex assay (LORR) was conducted as previously described (van der Linde et al., 2014). Approximately 30 male or female flies (2 to 5 days old) were placed in a clear plastic vial with a constant flow of EtOH/water vapor (1:1) delivered at 250 ml/min in each vial. At 5‐minute intervals, flies were assessed for their abilities to maintain postural control after a gentle tap. Flies that fell on their side or back for longer than 5 seconds were considered to have lost their righting reflex. The level of EtOH sensitivity was quantified as either the percentage of flies that have lost their righting reflex at a given time point or the amount of time that it took 50% of the flies to lose their righting as *t*_1/2_. All data were processed using Microsoft Excel, and the statistical significance was calculated using 1‐way or 2‐way ANOVAs followed by the Bonferroni post hoc test in the Statview program v5.0.1 (SAS Institute, Cary, NC) unless specified otherwise.

The LORR assay was used to measure the fly’s response to EtOH vapor for rapid tolerance (Berger et al., 2008; van der Linde et al., 2014). In the first exposure to EtOH vapor, the percentage of flies that lost their righting reflex was scored at 5‐minute intervals. After the indicated amount of exposure time, flies were kept in fresh food vials at 25°C incubator for 4 hours and tested again for LORR. The increase in LORR *t*_1/2_ between the 2 exposures was considered to be rapid tolerance.

### Fly Group Activity Monitor Assay

The fly group activity monitor (FlyGrAM) assay was set up and performed following a published protocol with slight modification (Scaplen et al., 2019). Specifically, 4 groups of 10 male flies were transferred into a 4‐chamber white acrylic arena with an overhead camera under dim light. The flies were allowed to acclimate for 20 minutes with a constant airflow at 125 ml/min to each chamber. The real‐time tracking of fly movement (as the number of moving flies) initiated at the rate of 30 frames per second under the following condition: 5‐minute airflow ‐>10‐minute EtOH vapor (1:1) ‐>5‐minute airflow. For data processing, the number of active flies was averaged in 10‐second bins using Microsoft Excel software. Flies that fell on their side or back at the end of the EtOH exposure were considered to be sedated and recorded. The average activity and sedated fly data were tested for normality using the Kolmogorov–Smirnov test. The statistical significance was then calculated with 1‐way ANOVA followed by the Bonferroni post hoc test or nonparametric Kruskal–Wallis test followed by Dunn’s multiple comparison test using the Statview program v5.0.1 (SAS Institute, Cary, NC).

### EtOH Absorption Assay

The whole‐head concentration of EtOH in flies was measured as described previously (Moore et al., 1998). For the naïve group, 30 male flies were exposed to EtOH/water (1:1) vapor as in the LORR assay for 10‐minute increments and immediately frozen in liquid nitrogen. For the preexposure group, 30 male flies were first exposed to EtOH/water vapor until 90% sedation in the LORR assay and let to recover on food at room temperature for 4 hours. Frozen flies were homogenized in 200*µ*l of 50 mM Tris‐HCl (pH 7.5) buffer on ice and centrifuged at 15,000 g for 20 minutes at 4°C. 1 *µ*l of clear supernatant from each sample was mixed with 19 *µ*l of 50 mM Tris‐HCl (pH 7.5) for dilution, and then, 2 *µ*l of diluted sample was mixed with 48 *µ*l of EtOH Assay Buffer (Sigma‐Aldrich, St. Louis, MO). The concentration of EtOH in each sample was calculated based on the increase in absorbance at 570 nm with various concentrations of EtOH (0, 2, 4, 6, 8, 10 nmole/ well) used as the standards. To measure the total protein concentration in flies, 1 *µ*l of cleared supernatant was mixed with 199 *µ*l of 50 mM Tris‐HCl (pH 7.5) for the preexposure group, and 1 *µ*l of cleared supernatant was mixed with 300 *µ*l of 50 mM Tris‐HCl (pH 7.5) for the Naïve group. 150 *µ*l of the diluted sample was incubated with 150 *µ*l 1× Dye Reagent (Bio‐Rad, Hercules, CA) for at least 5 minutes at room temperature. The final EtOH concentration is equal to (calculated EtOH concentration)/(total protein concentration).

### Immunostaining

Guinea pig anti‐GPRK2 antibody (Valanne et al., 2010) was obtained from David Hipfner (McGill University, QC). Rabbit polyclonal anti‐GFP antibody was purchased from Sigma‐Aldrich (St. Louis, MO). The 1D4 anti‐FasII monoclonal antibody was obtained from the Developmental Studies Hybridoma Bank (Iowa City, IA). Whole‐mount brain staining was done following a protocol described (Wu and Luo, 2006) with some modifications. Brains were dissected in PBS and fixed in 4% formaldehyde in PBS for 20 minutes on a rotator at RT. After fixation, the brains were washed in PBT (PBS with 0.3% Triton X‐100) quickly for 3 times, followed by 3 additional 20‐min washes. Subsequently, brains were incubated in the blocking solution (5% normal goat serum in PBT) overnight at 4°C. Primary antibodies were added to the blocking solution at the dilution of 1:400 (anti‐GPRK2) or 1:400 (anti‐FasII) where brains were incubated for 36 hours, followed by 20‐min washes in PBT for 5‐6 times. Then, the brains were incubated with secondary antibodies (anti‐guinea pig 594 nm and anti‐rabbit 488 nm, Thermo Fisher Scientific, Waltham, MA) at the dilution of 1:500 in blocking solution for 36 hours. Lastly, the brains were washed with PBT for 5 to 6 times with 20 minutes each before immersion in the Vectashield® mounting medium (Vector Laboratories, Burlingame, CA) overnight. The brains were then mounted and sequentially scanned using a Leica SP8 confocal microscope.

### Locomotion and Sleep Assay

Each 2‐ to 5‐day‐old male fly was housed individually in *Drosophila* Activity Monitors (Model DAM2 for 5‐mm tube; TriKinetics Inc., Waltham, MA) in a 25°C incubator with a 12‐hour/12‐hour light/dark cycle. Fly tubes contained 1.25% bactoagar with 5% sucrose at one end and were plugged with cotton at the other end. The DAMSystem software (v3.08; TriKinetics Inc.) tracked activity as the number of times a fly crossed an infrared beam through the center of the tube in 5‐minute bins. Five minutes without crossing the center was considered sleep (Hendricks et al., 2000; Shaw et al., 2000). Three days of data were collected after 2 days of entrainment to the light:dark cycle and analyzed using Microsoft Excel and R (R Development Core Team, 2015) to calculate sleep duration and bout number/length. For bout length and number, the Shapiro–Wilk normality test was performed, followed by the Kruskul–Wallis test for multiple‐group comparisons, using the XL‐Stat program (Addinsoft, New York, NY). For requesting the R script, please contact the author.

## Results

### *Gprk2* Mutants Show Reduced Naïve Sensitivity to EtOH

**Fig. 1:**
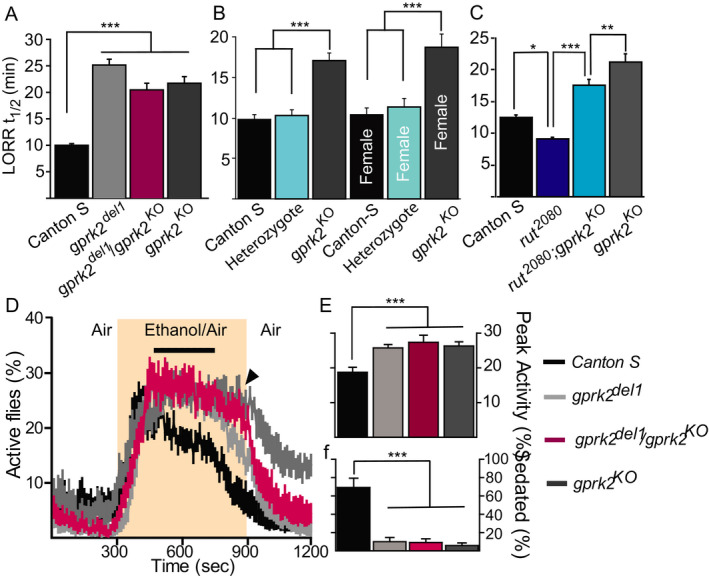
*Gprk2* mutants show decreased sensitivity to alcohol sedation but increased alcohol‐induced hyperactivity. (**A–C**) The loss of righting reflex assay was used to measure the amount of time that it took 50% of flies to lose posture control (LORR t_1/2_). Flies harboring different combinations of the *gprk2^del1^ or gprk2^KO^* amorphic alleles were compared to the wild‐type control (*Canton‐S*.* CS;* in a&b) and tested for their interaction with the *rutabaga* pathway (in C). *n* = 6 to 10. (**D–F**) The FlyGrAM assay was used to monitor real‐time locomotive activities (percentage of active flies) of *Gprk2* mutants with ethanol vapor delivered at 300 to 900 seconds (shaded). There was significant interaction between genotype and time as tested by the 2‐way ANOVA test, *p* < 0.0001 *F*(354, 5192) = 4.10 in **D**). The 4‐minute peak activities (black line in D&E) and percentage of sedated flies (f) at the end of alcohol exposure (arrowhead in d) were averaged and compared between *Canton‐S* flies (black) and *Gprk2* mutants (gray or magenta). *n* = 12. Mean ± SEM. *p < 0.05, **p < 0.01, ***p < 0.001 according to the 1‐way ANOVA with a Bonferroni post hoc test (**A–C, E**) or Kruskal–Wallis test followed by the Dunn’s multiple comparison test (**F**).

We examined mutants for the amorphic *gprk2^del1^* and *gprk2^KO^* alleles for differences in EtOH sensitivity from wild‐type flies. The *gprk2^del1^* is a small deficiency that removes 4 genes including *gprk2*, while the *gprk2^KO^* is a targeted knockout of *gprk2*, in which most of the open reading frame that encodes the kinase domain has been replaced with a mini‐*white* marker (Cheng et al., 2010). Both *gprk2^del1^* and *gprk2^KO^* homozygotes showed a reduced sensitivity to the intoxicating effects of EtOH compared to wild‐type flies in the loss of righting reflex (LORR) assay, *F*(3, 27) = 42.64, *p* < 0.001, Fig. 1 *A*. The *t*_1/2_ of *gprk2* mutants was significantly longer than that of *Canton‐S* flies (Fig. 1 *A*), suggesting a decreased sensitivity to EtOH. The *gprk2^del1^*/*gprk2^KO^* trans‐heterozygotes also showed a similar level of sensitivity as the *gprk2^KO^* mutant, suggesting that the reduced sensitivity was caused by the loss of *gprk2* function (*p* = 0.45, Fig. 1 *A*). Heterozygotes of wild‐type and *gprk2^KO^* alleles had alcohol sensitivity similar to *Canton‐S*, suggesting haplosufficiency of the wild‐type allele, *F*(5, 46) = 16.18, *p* = 0.7, Fig. 1 *B*. The same effect of *gprk2^KO^* on alcohol sensitivity was also observed in females, suggesting that the phenotype is not sex‐specific (*p* < 0.001, asterisks in Fig. 1 *B*). However, we used only male flies for the rest of the study unless specified.

To validate our observation that *Gprk2* mutants had altered responses to EtOH, we adopted the FlyGrAM assay to monitor fly’s real‐time locomotive activity during alcohol exposure (shaded in Fig. 1 *D*), which captured and separated the 3 stages of acute responses to alcohol: the initial startle response, the sustained hyperactivity, and the sedation (Scaplen et al., 2019; Wolf et al., 2002). A sustained and plateaued activity phase following the initial startle‐induced activity corresponded to a hyperactive state due to the stimulating effect of alcohol (Fig. 1 *D*) and was shown to be independent of the concentration of EtOH vapor. Therefore, we picked 4 minutes in the middle of the plateaued line to calculate averaged peak activity (black line in Fig. 1 *D*). Over time the flies became sedated, either seen as a decline in active flies or measured as the count of sedated flies at the end of alcohol exposure (arrowhead in Fig. 1 *D*). Compared to the wild‐type control, *Gprk2* mutant flies had increased alcohol‐induced hyperactivity, *F*(3, 44) = 7.12, *p* < 0.001 in Fig. 1 *E*, with significantly higher peak activity and decreased sensitivity to alcohol’s intoxicating effect with much fewer sedated flies (H_3_ = 20.16, *p* < 0.001 in Fig. 1 *F*). Therefore, from both LORR and FlyGrAM assays, we established that *Gprk2* was required for normal alcohol sensitivity. Furthermore, the decreased sensitivity to alcohol sedation found in the *Gprk2* mutants was not likely due to their differences in alcohol metabolism (i.e., lower alcohol concentration), since *Gprk2* mutant flies showed higher responses to the stimulating effect of alcohol.

The reduced sensitivity to EtOH sedation in *Gprk2* mutants was contrary to the expected outcome based on the regulatory role of GPRK2 on cAMP and Toll pathways in previous studies, suggesting a new mechanism for *Gprk2* in this context. Specifically, we examined *Gprk2* and *rutabaga (rut)* mutants for a genetic interaction in EtOH sensitivity. The *rut* gene encodes a type I adenylyl cyclase, and loss‐of‐function mutations in this gene lead to decreases in cAMP synthesis and increased EtOH sensitivity as indicated by a lower median elution time (MET) in the inebriometer (Levin et al., 1992a; Moore et al., 1998). Males with the loss‐of‐function *rut^2080^* allele displayed a significantly lower *t*_1/2_ in the LORR assay compared to *Canton‐S*, *F*(3, 32) = 34.88, *p* = 0.01, Fig. 1 *C*. Since *Gprk2* mutants were more resistant to alcohol, we speculated that *Gprk2* might reduce the activity of adenylyl cyclase by decreasing the levels of activated G(s)alpha through the agonist‐dependent desensitization of GPCRs. If *Gprk2* and *rut* are acting in the same pathway, then *rut^2080^* is predicted to be epistatic to the *gprk2^KO^* LORR phenotype. The *rut^2080^; gprk2^KO^* double mutants show an intermediate level of EtOH sensitivity, significantly different from that of *rut^2080^* (*p* < 0.0001, Fig. 1 *C*) and *gprk2^KO^* (*p* = 0.008, Fig. 1 *C*). However, we did not see a complete suppression of *Gprk2* mutant phenotype, suggesting that *Gprk2* is not affecting alcohol sensitivity solely through *rutabaga*, but likely also through additional pathways.

### *Gprk2* is Required in Ellipsoid Body Neurons to Mediate EtOH‐Induced Behavior

**Fig. 2:**
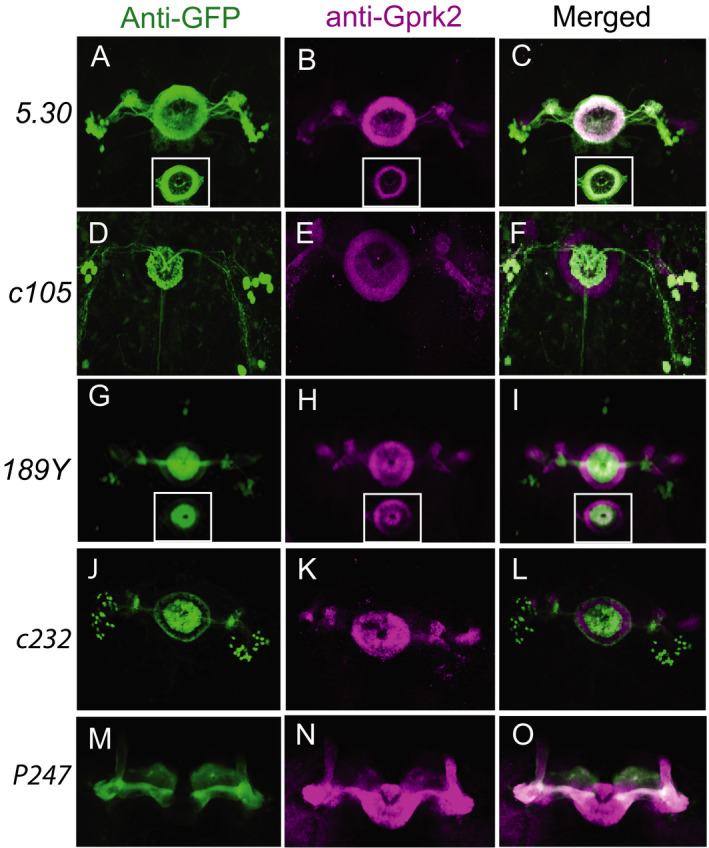
Immunostaining of GPRK2 protein (magenta) in ellipsoid body R2 and R4m neurons and mushroom body neurons in adult brains. Five Gal4 lines drive specific GFP expression in different sets of ellipsoid body neurons (**A** *5*.*30* in R2 and R4m neurons; **D** *c105* in R1 neurons; **G** *189Y* in R3 neurons; **J** *c232* in R3 and R4d neurons) or mushroom body neurons (m. *P247*) as shown in maximal projection images. Single confocal section images show complete overlap with 5.30 (inset in **A–C**) and partial overlap with 189y (inset in **G–I**).

*Gprk2* is preferentially expressed in the ellipsoid body (EB) and mushroom body (MB) neurons of the adult brain (Schneider and Spradling, 1997). We confirmed this expression pattern and further delimited *Gprk2* expression within the ellipsoid body using Gal4 lines that drive expression in different subsets of this neuropil (Renn et al., 1999; Fig. 2). GPRK2 is present in the R2 and R4m EB neurons as inferred by the complete overlap with GFP expression driven by *5*.*30* Gal4 (Fig. 2 *A*–*C*). Conversely, there are no or little overlaps between GPRK2 and GFP expression under the control of *c105*.* 189Y*. and *c232*, suggesting that GPRK2 is not strongly expressed in the R1 neurons (Fig. 2 *D*–*F*), R3 neurons (Fig. 2 *G*–*L*), or R4d neurons (Fig. 2 *J*–*L*), respectively. GPRK2 also shows strong expression in the alpha/beta and gamma mushroom body neurons as revealed by co‐expression with the MB specific driver *P247* (Fig. 2 *M*–*O*). Three additional EB Gal4 drivers, *c819*.* 4*.*67*. and *ruslanGal4*, have been reported to show preferential expression in R2 and R4m neurons (Kong et al., 2010a; Krashes and Waddell, 2008). Therefore, these Gal4 drivers were also used to study the functional requirement of GPRK2 in the R2 and R4m neurons.

**Fig. 3:**
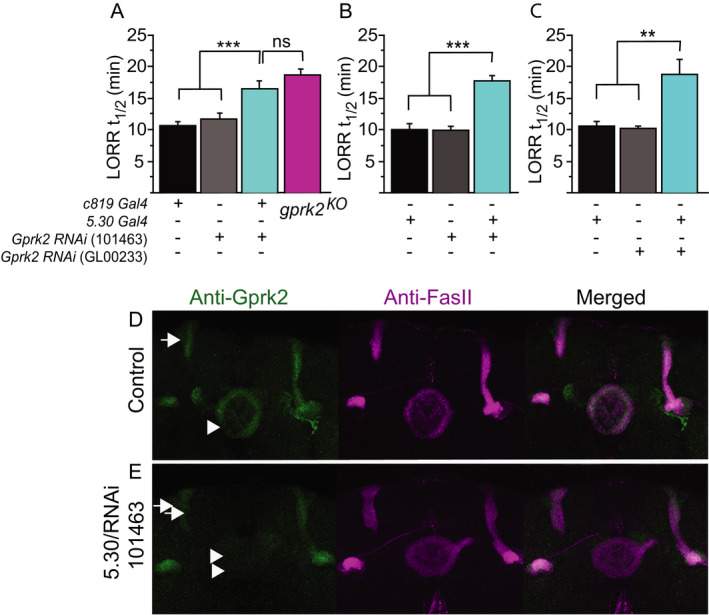
Knockdown of *Gprk2* expression in ellipsoid body neurons mimics *Gprk2* mutant phenotype in the LORR assay. (**A–C**). Two ellipsoid body Gal4 drivers, *c819* and *5*.*30*. were used to express 2 *Gprk2* RNA interference lines. Black/gray bars are control groups with Gal4 or UAS transgene only (*n* = 11 or 12). Cyan bars are experimental groups with both Gal4 and UAS transgene (*n* = 12 to 15). Open bars are *gprk2^KO^* mutants (*n* = 7). Mean ± SEM. **p < 0.01, ***p < 0.001 according to the 1‐way ANOVA with Bonferroni post hoc test. (**D**) Antibody staining of GPRK2 (green) in brains that express *Gprk2* RNAi using the EB‐specific driver 5.30. Anti‐FasII staining (purple) was used as counter staining to reveal mushroom bodies (arrows) and ellipsoid body (arrowheads).

Previous studies demonstrated that the EB played a prominent role in regulating naïve EtOH sensitivity (Rodan et al., 2002). We used the targeted expression of *Gprk2* RNAi transgenes to test whether *Gprk2* is required in ellipsoid body neurons to respond to EtOH. In control flies, GPRK2 is present in both MB and EB (Fig. 3 *D*). *Gprk2* protein level was reduced in EB neurons but remained unchanged in the MB neurons after the targeted expression of the *Gprk2* RNAi (101463) transgene in the R2‐R4M EB neurons (Fig. 3E). In contrast, Fasciclin II expression appeared to be the same in the *Gprk2* RNAi–expressing brains and the controls, with a strong expression in MB and weaker expression in EB (Fig. 3 *D*,*E*). These data suggest that MB and EB structures were not affected significantly by the developmental expression of *Gprk2* RNAi. We concluded that the *Gprk2 RNAi* line (*101463)* was sufficient to block *Gprk2* mRNA expression and could serve as a good tool to study the functional requirement of *Gprk2* in ellipsoid body neurons. An independently generated *RNAi* line targeting *Gprk2* (*GL00233*) was also used to validate our results.

We conducted the LORR assay with RNAi knockdown of *Gprk2* to further validate the role of this gene in regulating EtOH sensitivity. Control flies containing the Gal4 drivers and UAS‐RNAi transgenes displayed EtOH sensitivity similar to wild‐type flies (Fig. 3 *A*–*C*) as they showed the same level of sensitivity in the LORR assay. When the *UAS‐Gprk2 RNAi (101463)* was driven by the *c819 gal4 driver* in the R2‐R4M EB neurons, there was a significant increase in the LORR t_1/2_, suggesting reduced sensitivity to EtOH compared to the controls, *F*(3, 42 = 12.19, *p* < 0.001, Fig. 3 *A*. The severity of the phenotype was comparable to *gprk2^KO^* mutants (*p* = 0.17, Fig. 3 *A*). The same effect was also observed with *5*.*30* gal4 line driving the expression of *Gprk2 RNAi* line *101463*, *F*(2, 32) = 30.79, *p* < 0.0001, Fig. 3 *B*. Moreover, flies with the *5*.*30* gal4 driving the expression of *Gprk2 RNAi GL00233*, *F*(3, 44) = 23.40, *p* < 0.01 Fig. 3 *C* also displayed reduced EtOH sedation sensitivity, strongly suggesting the observed phenotype was specific to the loss of function of *Gprk2* in R2 and R4m neurons.

**Fig. 4:**
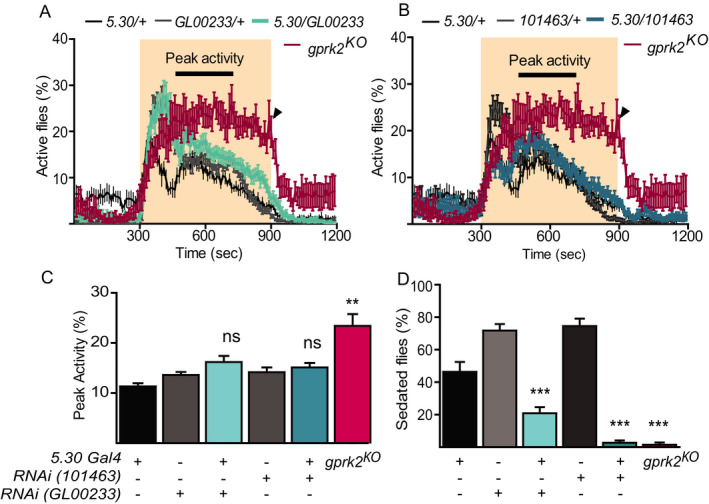
The decreased sensitivity to alcohol is not linked to increased alcohol‐induced hyperactivity when *Gprk2* is knocked down in EB neurons. All experimental (cyan in **A&B**) and control groups were tested at the same time but presented for each RNAi line separately in **A** and **B**. Disruption of *Gprk2* expression using 2 independent lines (*GL00233* and *101463*) with EB‐specific driver (*5*.*30*) showed no significant difference in 4‐minute peak activity (cyan in **C**) but decreased sensitivity to alcohol (cyan in **D**) compared to controls (black and gray in C&D). *gprk2^KO^* flies were used as positive controls (magenta, *n* = 7). *n* = 11 unless specified. Mean ± SEM. ns, *p* > 0.05, **p < 0.01, ***p < 0.001 according to the 1‐way ANOVA with Bonferroni post hoc test after the data passed the Kolmogorov‐Smirnov normality test.

Interestingly, *Gprk2* mutants not only have decreased sensitivity to alcohol’s sedating effect but also show higher EtOH‐induced hyperactivity (Fig. 1 *D*), which has also been mapped to EB neurons (Kong et al., 2010b). These shared genetic and neuronal requirements suggest EtOH‐induced sedation and hyperactivity may share a molecular process. Perhaps *Gprk2* mutant flies were over‐stimulated by EtOH and thus responded more slowly to its sedating effect. We further tested the idea of shared requirements using RNAi knockdown of *Gprk2* in EB neurons (Fig. 4). Similar to previous observations, we found high variability in the initial startle response to alcohol in flies with different transgenes (Scaplen et al., 2019). Therefore, we used the 4‐min peak activity at the plateaued lines, which is independent of alcohol concentration and out of the startled response phase, to measure EtOH‐induced hyperactivity (black lines in Fig. 4 *A*.*B*). Compared to control flies, *gprk2^KO^* mutants had higher peak activity, *F*(5, 56) = 11.3, *p* < 0.01, magenta in Fig. 4 *C*) and significantly fewer sedated flies, *F*(5, 56) = 61.50, *p* = 0.0001, magenta in Fig. 4 *D* at the end of the EtOH exposure. Compared to controls with either the Gal4 or the UAS transgene only (black and gray in Fig. 4 *D*), the expression of either RNAi line with the EB‐specific driver *5*.*30* resulted in significantly fewer sedated flies, *F*(5, 56) = 61.5, *p* < 0.0001, cyan in Fig. 4 *D*, suggesting decreased alcohol sensitivity. However, the RNAi treatment did not change their peak activity (*p* > 0.05, cyan in Fig. 4 *C*) compared to the controls with each transgene (black and gray in Fig. 4 *C*), suggesting that EtOH‐induced hyperactivity and EtOH sedation sensitivity are not phenotypically linked. In other words, the decreased sensitivity to EtOH’s sedating effect was not caused by an enhanced alertness state with EtOH’s stimulating effect. However, we could not rule out that *Gprk2* expression in EB neurons might be involved in both processes as they might have different thresholds to exhibit the phenotypes.

**Fig. 5:**
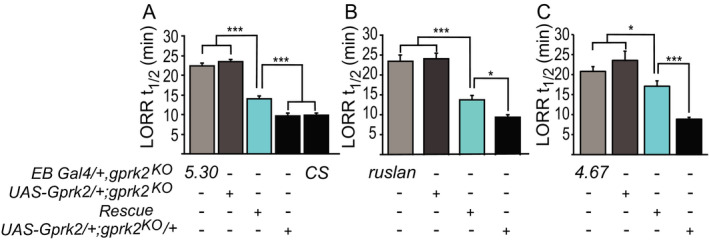
Expression of *Gprk2* transgene in ellipsoid body neurons rescues ethanol (EtOH) sensitivity in *Gprk2* mutant flies. (**A–C**) Loss of righting reflex (LORRt_1/2_) was used to measure the level of EtOH sensitivity in flies. Three ellipsoid body neuron Gal4 drivers, *5*.*30* (**A**), *ruslan* (**B**), and *4*.*67* (**C**), were used to target the expression of *Gprk2* in *gprk2^KO^* mutants. Open bars, Gal4 or UAS transgene alone in *gprk2^KO^* homozygous background; cyan bars, Gal4 and UAS transgene in *gprk2^KO^* homozygous background; black bars, *gprk2^KO^* heterozygous or wild‐type (CS) background. *n* = 10‐20. Mean ± SEM. *p < 0.05, **p < 0.01, ***p < 0.001 according to the 1‐way ANOVA with Bonferroni post hoc test.

We further expressed a *Gprk2* cDNA in the *gprk2^KO^* mutant background using the 3 EB‐specific *Gal4* drivers, *5*.*30*, *ruslanGal4*. and *4*.*67*. The UAS‐*Gprk2* transgene or Gal4 driver alone did not change the *gprk2^KO^* EtOH sensitivity phenotype (Fig. 5). Consistent with the haplosufficiency of *Gprk2* seen in Fig. 1 *B*, the EtOH sensitivity of *UAS‐Gprk2/+; gprk2^KO^/+* flies was indistinguishable from *Canton‐S* (CS) flies (*p* = 0.83). The *5*.*30*, *F*(4, 77) = 116.62, *p* < 0.0001, Fig. 5 *A*, and *ruslanGal4*, *F*(3, 41) = 37.42, *p* < 0.0001, Fig. 5 *B*, drivers significantly rescued the UAS‐*Gprk*2/+; *gprk2^KO^* sensitivity phenotype with high Cohen’s effect size values, *d* = 15.81 and 8.4, respectively), while the *4*.*67* driver produced a significant rescue, *F*(3, 29) = 23.09, *p* < 0.0001, Fig. 5 *C*, with moderate effect (*d* = 3.65). Specifically, rescue with *4*.*67* flies was closer to the mutant controls (*4*.*67* control, *p* = 0.04 and *UAS‐Gprk2* control, *p* = 0.003, Fig. 5 *C*) than to the wild‐type control (*p* < 0.0001, Fig. 5 *C*). Compared to 5.30 and *ruslanGal4*, *4*.*67* has relatively weak expression in the EB (Rodan et al., 2002). Therefore, the differences in the rescue experiment may reflect the magnitude of expression of the 3 EB drivers.

### *Gprk2* is Required for Rapid Tolerance to Alcohol

Several fly mutants with altered EtOH sensitivity also show changed levels of rapid tolerance, suggesting common mechanisms between the initial response to EtOH and the physiological changes that are responsible for rapid tolerance even though no correlation was found between the 2 phenotypes (Berger et al., 2008; Devineni et al., 2011). Therefore, we wanted to see whether *Gprk2* was also required for rapid tolerance.

**Fig. 6:**
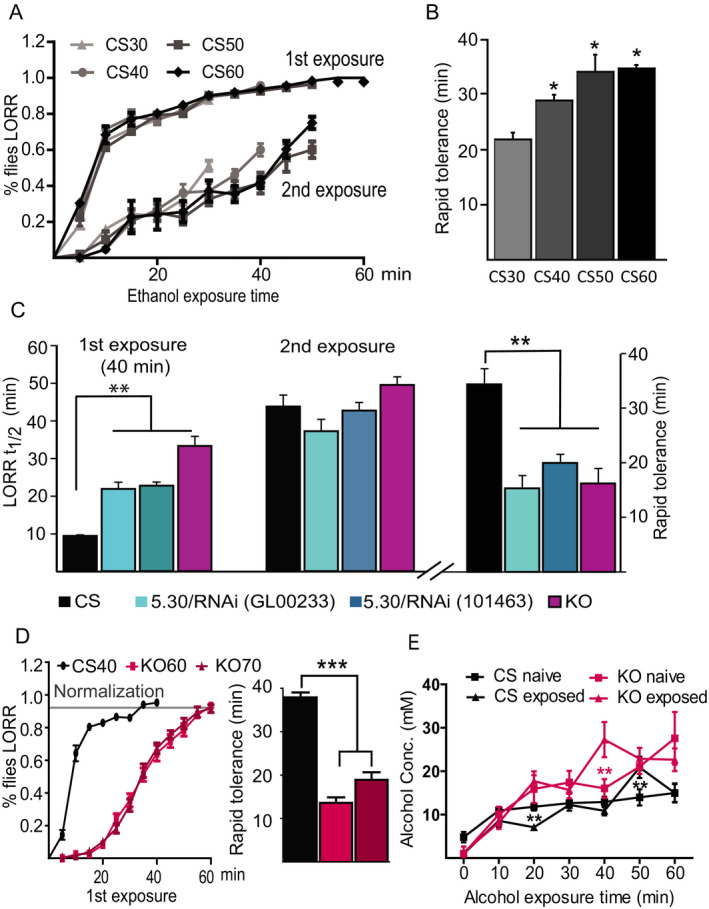
*Gprk2* loss of function affects rapid tolerance as measured in the LORR assay. (**A‐B**) Wild‐type (CS) flies developed rapid tolerance 4 hours after exposure to EtOH for 30 minutes (CS30), 40 minutes (CS40), 50 minutes (CS50), or 60 minutes (CS60). The difference in LORR t_1/2_ between the 2 exposures (second minus first) is rapid tolerance. *p < 0.05. *n* = 4. (**C**) Wild‐type (CS), *gprk2^KO^* mutants, and RNAi‐expressing flies (*5*.*30/RNAi (GL00233)* or *5*.*30/RNAi (101463))* were tested for LORR *t*_1/2_ for 40 minutes (first exposure) and 4 hours after (second exposure). *n* = 4. **p < 0.001. (**D**) Wild‐type (*CS40*) flies were exposed to EtOH for 40 min, while *gprk2^KO^* flies were exposed to 60 minutes (*KO60*) and 70 minutes (*KO70*), respectively, until more than 90% of the flies lost their righting. The rapid tolerance was assessed 4 hours later. *n* = 12. ***p < 0.0001. (**E**) The whole‐head alcohol concentration was measured in wild‐type (*CS*) and *gprk2^KO^* (*KO*) flies when exposed to 50% alcohol vapor for 10, 20, 30, 40, 50, and 60 minutes, respectively. Naïve flies of different genotypes (CS and KO) were compared at each time point (CS naïve vs. KO naïve at 40, 50, 60 minutes, *p* < 0.001***; CS exposed vs. KO exposed at 20, 40 minutes, *p* < 0.01** and *p* < 0.001***). In addition, the alcohol concentration between naïve and exposed flies for both genotypes was compared at each time point (CS naïve vs. CS exposed, KO naïve vs. KO exposed, *p* > 0.05). The 2‐way ANOVA Bonferroni/Dunn tests were used in the statistical analysis. *n* = 6 for each genotype and treatment. *F*(3, 164) = 24.48 for genotype/treatment, *F*(6, 161) = 41.14 for exposure time.

We first tested the rapid tolerance protocol on *Canton‐S* flies using the LORR paradigm. When exposed to EtOH for 30, 40, 50, or 60 minutes, flies displayed visually similar curves plotting the percentile of flies losing their righting reflex in the function of EtOH exposure time (Fig. 6 *A*). Upon the second exposure, 4 hours later, flies showed a notably delayed response to EtOH, suggesting the development of rapid tolerance similar to what was reported previously (Fig. 6 *A*&*B*). There was a significant increase in rapid tolerance from 30 minutes, which stabilizes at 40, 50, and 60 minutes of EtOH exposure (40 minutes, *p* = 0.01; 50 and 60 min, *p* < 0.001; *F*(3, 12) = 11.7, Fig. 6 *B*). Noticeably, when exposed to EtOH for 60 minutes, we found that a significant number of flies never recovered after 4 hours. We, therefore, used 40 minutes in the first exposure for testing rapid tolerance in future experiments.

When exposed to EtOH for 40 minutes in the first trial, *gprk2^KO^* flies had significantly lower levels of rapid tolerance compared to wild‐type flies when tested 4 hours later, *F*(3, 12) = 13.06, *p* = 0.0002, Fig. 6 *C*. The same phenotype was observed in *Gprk2 RNAi*–expressing flies using *5*.*30 Gal4* driver in comparison with the wild‐type control (*p* = 0.0001 and *p* = 0.0013, respectively). At 40 minutes of the first exposure, 95% *Canton‐S* flies were sedated, while approximately 65% of *gprk2^KO^* flies lost their posture control (Fig. 6 *D*). To compensate for this difference, we decided to increase the exposure time for *gprk2^KO^* flies to 60 and 70 minutes until 95% of the flies also lost their righting reflex, just as wild‐type flies behaved with 40‐minute exposure to EtOH (Fig. 6 *D*). At a higher dosage of EtOH vapor, *gprk2^KO^* flies still failed to develop the same level of rapid tolerance as wild‐type flies, *F*(2, 33) = 82.07, *p* < 0.0001, Fig. 6 *D*. Compared to the wild‐type control, *gprk2^KO^* flies displayed approximately 50% of the level of rapid tolerance with an increasing amount of exposure time at 40, 60, and 70 minutes, suggesting that the deficiency was likely due to a lack of functional rapid tolerance development rather than insufficient exposure to alcohol. Two more observations supported this interpretation. First, *gprk2^KO^* mutant flies showed higher alcohol concentration compared to wild‐type flies according to the 2‐way ANOVA test, *F*(3, 20) = 24.48, *p* < 0.01 at 20 minutes, *p* < 0.001 at 40, 50, 60 minutes, asterisks in Fig. 6 *E*. Secondly, neither the wild‐type nor *Gprk2* mutant flies showed significant changes in how they metabolized alcohol before and after alcohol exposure (*p* > 0.05 for all CS‐naïve vs. CS‐exposed groups or KO‐naïve vs. KO‐exposed groups in Fig. 6 *E*). It was previously shown that water vapor itself can elicit a decreased sensitivity to EtOH in flies between the first and second exposure (Scholz et al., 2000). Therefore, the residual rapid tolerance in *gprk2^KO^* flies seen in our experiment may be nonspecific changes generated by other factors. In summary, these data suggest that *Gprk2* is not only required for a fly’s acute response to EtOH but also to develop rapid tolerance to EtOH after initial exposure.

### *Gprk2*‐Dependent Alcohol Response Does not Correlate With State of Alertness

**Fig. 7:**
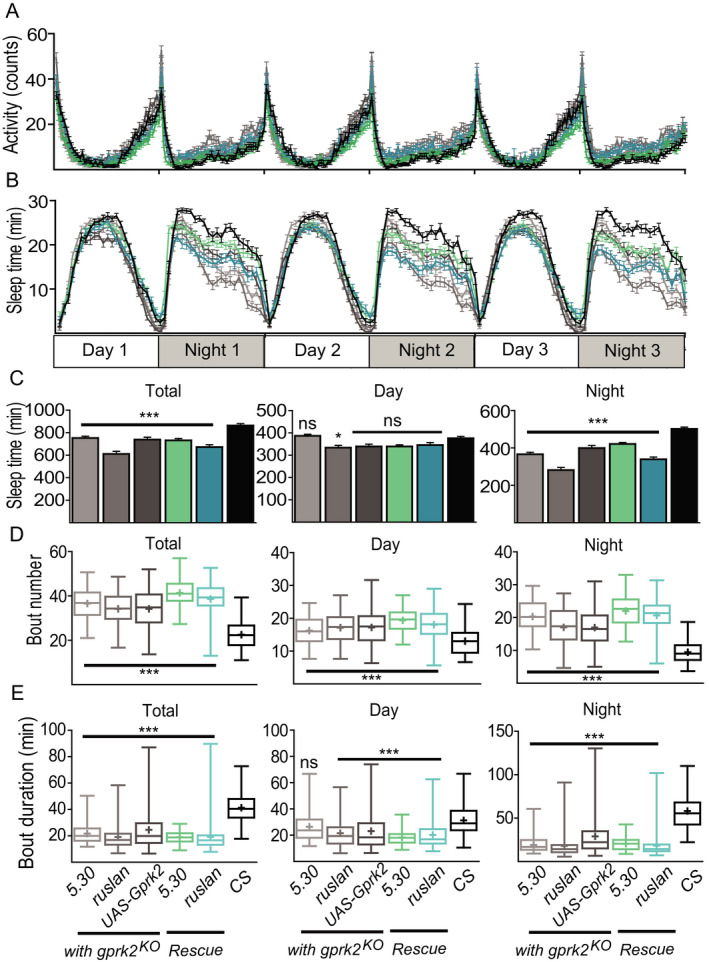
Expression of *Gprk2* in ellipsoid body neurons does not rescue the fragmented sleep patterns in *Gprk2* knockout flies. *Canton‐S* (black), *gprk2^KO^* rescue flies with 2 different Gal4 drivers (*5*.*30 Rescue* and *ruslan Rescue*, cyan), *gprk2^KO^* control flies with either the Gal4 drivers or UAS transgene only (*5*.*30 KO*.* ruslan KO*.* UAS‐Gprk2 KO*, gray) showed similar levels of locomotive activity (**A**) but decreased sleep time, specifically during night (**B**, **C**). The sleep time (**C**), bout number (**D**), and duration (**E**) were measured and compared between Rescue or *gprk2^KO^* mutant control flies and the wild‐type control (*Canton‐S*). For respective total, day and night sleep time, *F*(5, 506) = 19.08, 5.779, 41.33. For the respective total, day and night bout number, H_5_ = 184.3, 83.49, 200. For the respective total, day and night bout duration, H_5_ = 164.4, 76.55, 196.4. One‐way ANOVA test followed by Bonferroni post hoc (**C**) or Kruskal–Wallis tests followed by Dunn multiple comparison post hoc (**D, E**) were used in the statistical analysis. Means are indicated by + in each box. *n* = 77 to 88 male flies for each genotype. ns, *p* > 0.05, *p < 0.05, ***p < 0.001.

Flies display a rhythmic 24‐hour cycle of locomotive activity (Fig. 7 *A*) and sleep pattern (Fig. 7 *B*), subject to circadian control. We wondered whether there is a link between the lower sensitivity to alcohol in *Gprk2* mutants and their states of wakefulness and alertness. Such a link predicts that *Gprk2* mutants have alterations in their locomotive and sleep patterns, which can be rescued with targeted expression of *Gprk2* in EB neurons. Compared to the wild‐type control (black in Fig. 7), *gprk2^KO^* mutants with *5*.*30*.* ruslanGal4*. or *UAS‐Gprk2* transgene alone (gray in Fig. 7) did not show significant changes in their overall locomotion activities (Fig. 7 *A*), but significant loss in overall sleep during nights (*p* < 0.001, Fig. 7 *C*). We further observed sleep fragmentation in *Gprk2* mutant groups with more frequent shorter sleep bouts in both days and nights, suggesting a change in sleep quality (*p* < 0.001, Fig. 7 *D*.*E*). However, this mutant phenotype in sleep was not rescued when *Gprk2* was expressed in EB neurons (cyan in Fig. 7 *A*–*E*). We concluded that even though *Gprk2* may be required for basic sleep patterns in flies, this requirement is not mediated by EB neurons. Therefore, the EB‐dependent requirement of *Gprk2* for normal alcohol sensitivity and tolerance does not correlate directly with animals’ state of alertness or locomotive activity.

## Discussion

The GRK family regulates GPCRs through agonist‐dependent receptor trafficking and internalization. Our study has revealed a new role for *Gprk2* in EtOH‐induced behaviors in *Drosophila*. There is a positive genetic interaction between GPRK2 activity and cAMP signaling in egg morphogenesis and wing development (Cheng et al., 2012; Lannutti and Schneider, 2001, p. 2). Moreover, cAMP signaling through the *rutabaga* adenylyl cyclase has an established role in modulating EtOH sedation (Rodan et al., 2002). Nevertheless, we have found that GPRK2’s role in EtOH sedation is independent of *rutabaga*‐dependent cAMP signaling.

Furthermore, we mapped out the requirement of *Gprk2* in EtOH sedation sensitivity to the ellipsoid body neurons, suggesting that *Gprk2* is required for the neuronal responses to EtOH. The formation of rapid tolerance is also diminished in the absence of *Gprk2*. This result suggests that agonist‐dependent desensitization, which is responsible for signal gain control, modifies the homeostatic processes involved in tolerance formation. The availability of *Gprk2* to maintain normal regulation of GPCRs is essential to maintain the physiological state of EB neurons (normal level of locomotive activity) and confers plasticity that is required to develop rapid tolerance to alcohol.

### The Role for *Gprk2* in EtOH Sedation Sensitivity is Largely Independent of cAMP Signaling

Studies have suggested that G protein–coupled receptor kinases interact with G protein–coupled receptors and regulate downstream signaling through different mechanisms (Cheng et al., 2012; Evron et al., 2012; Hanlon and Andrew, 2015; Topalidou et al., 2017). In the canonical model, GRKs function by phosphorylating G protein–coupled receptors upon ligand binding and desensitize surface receptors through β‐arrestin‐mediated receptor internalization. Therefore, a simple prediction is that loss of GRK activity would result in the continuing activation of receptors, and prolong their effects on downstream signaling cascades.

The cAMP signaling pathway is one effector system that would be affected by a loss in *Gprk2*. This pathway is important for many neurological processes including arousal/sleep, learning and memory, and alcohol‐induced behaviors (Davis et al., 1995; Hendricks et al., 2001; Wand et al., 2001; Yang et al., 2003). Not surprisingly, GPRK2 regulates the cAMP level in a tissue‐specific manner; for example, GPRK2 positively regulates cAMP levels in both egg morphogenesis and wing development (Cheng et al., 2012; Schneider and Spradling, 1997), yet in developing wing disks, GPRK2 downregulates Smoothened (Smo) in response to Hedgehog (Cheng et al., 2012). Smo acts through the inhibitory G(i)alpha, which inhibits adenylyl cyclase, reducing cAMP levels (Ogden et al., 2008). Hence, the impact of GPRK2 activity on cAMP signaling would be predicted to be widespread and context‐dependent.

In the *rut^2080^* mutant, cAMP levels are slightly decreased (Levin et al., 1992b). In neurons expressing *rut*, the loss‐of‐*Gprk2* function may result in higher cAMP levels due to the loss of G protein–coupled receptor desensitization, opposite to the phenotype of the *rut* mutant. The *rut* mutant has a higher sensitivity to alcohol, whereas the *Gprk2* mutant showed a decreased sensitivity, consistent with this model. However, *rut^2080^* was not epistatic to *gprk2^KO^* in EtOH sedation as expected, if *Gprk2* mostly or solely acts upstream of *rut* in this context. The *rut^2080^; gprk2^KO^* double mutant has an additive phenotype, suggesting that additional signaling pathways or mechanisms also contribute to the *gprk2^KO^* reduced EtOH sensitivity phenotype. This model of *Gprk2* acting through cAMP‐independent pathways is further supported by the observation that inhibition of PKA activity in EB did not affect EtOH sensitivity (Rodan et al., 2002).

### *Gprk2* Acts Within Ellipsoid Body Neurons to Modulate EtOH Sedation Sensitivity

The EB neurons are involved in several locomotion‐related behaviors, including walking, turning, visual orientation, and sleep drive (Guo et al., 2014; Kottler et al., 2019;; Robie et al., 2017). Consistently, disruptions to the structural integrity or physiological states of these neurons alter animals’ locomotion‐dependent responses to alcohol (Kong et al., 2010; Scaplen et al., 2019; Urizar et al., 2007). We have mapped a major functional requirement for *Gprk2* in alcohol sedation sensitivity and rapid tolerance to the EB R2‐R4m neurons, but this site is not a focus for its requirement for the alcohol‐induced hyperactivity or sleep. The latter 2 functions are known to be mediated through dopaminergic neurons that control sleep/wake cycles outside EB (Kong et al., 2010a; Ly et al., 2018). Therefore, GPRK2 may affect EtOH behaviors and arousal through dopamine signaling pathways outside of the EB. One possible signaling pathway for GPRKs within the EB neurons involves octopamine GPCR signaling. Flies with low octopamine levels display increased EtOH sensitivity and fail to develop rapid tolerance (Scholz, 2005; Scholz et al., 2000). Alternatively, GPRK2 has also been shown to increase neurotoxicity by phosphorylating the human α‐synuclein in a *Drosophila* model of Parkinson’s disease (Chen and Feany, 2005), suggesting a GPCR‐independent mechanism.

Another possible signaling pathway for *Gprk2* in EtOH sedation would include the mitogen‐activated protein kinases/extracellular signal‐regulated kinases (MAPK/ERK) pathway. In mice, β‐arrestin 2 works upstream of ERK signaling and β‐arrestin 2 knockout mice have reduced sensitivity to alcohol, similar to the loss‐of‐function phenotype in *gprk2^KO^* (Li et al., 2013). In *Drosophila*, the only β‐arrestin, Kurtz, also regulates MAPK/ERK signaling by binding to and sequestering the inactive form of ERK during embryo development (Tipping et al., 2010). This interaction was confirmed and could be triggered with the activation of GPCRs as shown *in vitro* (Eishingdrelo et al., 2015). To further support this model, ERK is downstream of EGFR pathway, which has been shown to mediate EtOH‐induced behaviors (Corl et al., 2009; King et al., 2014).

### *Gprk2* and Neuronal Plasticity

Flies develop rapid tolerance to alcohol 4 hours after a single exposure as the function of neural plasticity (Rodan and Rothenfluh, 2010; Scholz et al., 2000). EtOH sensitivity and rapid tolerance are genetically separable, suggesting distinct pathways might be responsible (Berger et al., 2008; Devineni et al., 2011). For instance, mutants of *hangover* had normal sensitivity to alcohol but reduced rapid tolerance (Scholz et al., 2005, p. 200). Flies without Homer, a postsynaptic scaffolding protein in regulating synaptic structure and/or plasticity, had increased sensitivity to alcohol and failed to develop rapid tolerance (Urizar et al., 2007). Our work showed that *Gprk2* mutants had decreased sensitivity to alcohol and significantly reduced the ability to develop rapid tolerance, suggesting a role for *Gprk2* in neuronal plasticity.

For mutants that have different naïve sensitivity to alcohol, measurement of rapid tolerance can be confounded by the difference in the initial neuronal state. In our experiment, we modified the existing training paradigm where different exposure times were given to each group of flies until 95% of flies were sedated. This modification allowed us to separately assess the sensitivity and rapid tolerance phenotype. Despite the prolonged exposure to EtOH, *Gprk2* mutant flies never developed the same level of rapid tolerance as wild‐type flies, suggesting that the signaling pathway responsible for rapid tolerance is disrupted in *Gprk2* mutants. As suggested by the work on GRK2 in mice, exposure to EtOH can increase GRK binding and phosphorylation to GPCR (Zhang et al., 1998). Without GRKs, neurons may no longer be able to adapt by adjusting the amount or activity of GPCRs at synapses. Therefore, mutant flies fail to develop tolerance to alcohol and act like naïve flies despite previous exposure to EtOH. Alternatively, upregulation of GPCR amount or activity at synapses is a key mechanism of rapid tolerance to alcohol, and therefore, *Gprk2* mutant flies may resemble “preexposed” flies due to net gain in GPCR activities without *Gprk2*‐dependent receptor internalization.

### Common Mechanism With Vertebrates

*Drosophila* GPRK2 shows functional homology to its divergent mammalian counterparts in the GRK4/5/6 subfamily. Specifically, GPRK2 and GRK5 are both involved in regulating the NF‐κB pathway and possibly protecting from neurodegeneration (Arawaka, 2006; Chen and Feany, 2005; Valanne et al., 2010). GRK6 has been linked to the dopamine signaling pathway in a mouse Parkinson’s disease model (Managò et al., 2012). The same link can be drawn for *Drosophila Gprk2*, whose loss of function leads to altered EtOH‐induced hyperactivity and sleep patterns, both of which are dopamine‐dependent (Kong et al., 2010a; Ly et al., 2018). It has yet to be seen whether members of the vertebrate GRK4/5/6 subfamily function in modulating EtOH sensitivity and rapid tolerance formation.

## Conflict of Interest

There is no conflict of interest in this study.

## References

[acer14396-bib-0001] Arawaka S (2006) The role of G‐protein‐coupled receptor kinase 5 in pathogenesis of sporadic Parkinson’s disease. J Neurosci 26:9227–9238. 16957079 10.1523/JNEUROSCI.0341-06.2006 PMC6674490

[acer14396-bib-0002] Berger KH , Kong EC , Dubnau J , Tully T , Moore MS , Heberlein U (2008) Ethanol sensitivity and tolerance in long‐term memory mutants of *Drosophila* melanogaster. Alcohol Clin Exp Res 32:895–908. 18435628 10.1111/j.1530-0277.2008.00659.x PMC3044939

[acer14396-bib-0004] Cassill JA , Whitney M , Joazeiro CA , Becker A , Zuker CS (1991) Isolation of *Drosophila* genes encoding G protein‐coupled receptor kinases. Proc Natl Acad Sci USA 88:11067–11070. 1662381 10.1073/pnas.88.24.11067 PMC53074

[acer14396-bib-0005] Chen L , Feany MB (2005) α‐Synuclein phosphorylation controls neurotoxicity and inclusion formation in a *Drosophila* model of Parkinson disease. Nat Neurosci 8:657–663. 15834418 10.1038/nn1443

[acer14396-bib-0006] Cheng S , Maier D , Hipfner DR (2012) *Drosophila* G‐protein‐coupled receptor kinase 2 regulates cAMP‐dependent Hedgehog signaling. Development 139:85–94. 22096079 10.1242/dev.068817

[acer14396-bib-0007] Cheng S , Maier D , Neubueser D , Hipfner DR (2010) Regulation of smoothened by *Drosophila* G‐protein‐coupled receptor kinases. Dev Biol 337:99–109. 19850026 10.1016/j.ydbio.2009.10.014 PMC3160985

[acer14396-bib-0008] Corl AB , Berger KH , Ophir‐Shohat G , Gesch J , Simms JA , Bartlett SE , Heberlein U (2009) Happyhour, a Ste20 family kinase, implicates EGFR signaling in ethanol‐induced behaviors. Cell 137:949–960. 19464045 10.1016/j.cell.2009.03.020

[acer14396-bib-0009] Davis RL , Cherry J , Dauwalder B , Han PL , Skoulakis E (1995) The cyclic AMP system and *Drosophila* learning. Mol Cell Biochem 149–150:271–278. 10.1007/978-1-4615-2015-3_31 8569740

[acer14396-bib-0010] Development Core Team R (2015) R: A Language and Environment for Statistical Computing R Foundation for Statistical Computing, Vienna, Austria.

[acer14396-bib-0011] Devineni AV , McClure KD , Guarnieri DJ , Corl AB , Wolf FW , Eddison M , Heberlein U (2011) The genetic relationships between ethanol preference, acute ethanol sensitivity, and ethanol tolerance in *Drosophila* melanogaster. Fly (Austin) 5:191–199. 21750412 10.4161/fly.5.3.16987 PMC3225762

[acer14396-bib-0012] Dietzl G , Chen D , Schnorrer F , Su K‐C , Barinova Y , Fellner M , Gasser B , Kinsey K , Oppel S , Scheiblauer S , Couto A , Marra V , Keleman K , Dickson BJ (2007) A genome‐wide transgenic RNAi library for conditional gene inactivation in *Drosophila* . Nature 448:151–156. 17625558 10.1038/nature05954

[acer14396-bib-0013] Edenberg HJ , Foroud T (2014) Genetics of alcoholism. Handb Clin Neurol 125:561–571. 25307596 10.1016/B978-0-444-62619-6.00032-X

[acer14396-bib-0014] Eishingdrelo H , Sun W , Li H , Wang L , Eishingdrelo A , Dai S , McKew JC , Zheng W (2015) ERK and β‐arrestin interaction: a converging point of signaling pathways for multiple types of cell surface receptors. J. Biomol. Screen. 20:341–349. 25361946 10.1177/1087057114557233 PMC4975872

[acer14396-bib-0015] Evron T , Daigle TL , Caron MG (2012) GRK2: multiple roles beyond G protein‐coupled receptor desensitization. Trends Pharmacol Sci 33:154–164. 22277298 10.1016/j.tips.2011.12.003 PMC3294176

[acer14396-bib-0016] Fukuto HS , Ferkey DM , Apicella AJ , Lans H , Sharmeen T , Chen W , Lefkowitz RJ , Jansen G , Schafer WR , Hart AC (2004) G protein‐coupled receptor kinase function is essential for chemosensation in C. elegans. Neuron 42:581–593. 15157420 10.1016/s0896-6273(04)00252-1

[acer14396-bib-0017] Ghezzi A , Al‐Hasan YM , Krishnan HR , Wang Y , Atkinson NS (2013) Functional mapping of the neuronal substrates for drug tolerance in *Drosophila* . Behav Genet 43:227–240. 23371357 10.1007/s10519-013-9583-0 PMC4586076

[acer14396-bib-0018] Grotewiel M , Bettinger JC (2015) *Drosophila* and *Caenorhabditis elegans* as discovery platforms for genes involved in human alcohol use disorder. Alcohol Clin Exp Res 39:1292–1311. 26173477 10.1111/acer.12785 PMC4656040

[acer14396-bib-0019] Guo C , Du Y , Yuan D , Li M , Gong H , Gong Z , Liu L (2014) A conditioned visual orientation requires the ellipsoid body in *Drosophila* . Learn Mem Cold Spring Harb N 22:56–63. 10.1101/lm.036863.114 PMC4274327 25512578

[acer14396-bib-0020] Hanlon CD , Andrew DJ (2015) Outside‐in signaling ‐ a brief review of GPCR signaling with a focus on the *Drosophila* GPCR family. J Cell Sci 128:3533–3542. 26345366 10.1242/jcs.175158 PMC4610211

[acer14396-bib-0021] Hendricks JC , Finn SM , Panckeri KA , Chavkin J , Williams JA , Sehgal A , Pack AI (2000) Rest in *Drosophila* is a sleep‐like state. Neuron 25:129–138. 10707978 10.1016/s0896-6273(00)80877-6

[acer14396-bib-0022] Hendricks JC , Williams JA , Panckeri K , Kirk D , Tello M , Yin JC , Sehgal A (2001) A non‐circadian role for cAMP signaling and CREB activity in *Drosophila* rest homeostasis. Nat Neurosci 4:1108–1115. 11687816 10.1038/nn743

[acer14396-bib-0023] Homan KT , Tesmer JJG (2014) Structural insights into G protein‐coupled receptor kinase function. Curr Opin Cell Biol 27:25–31. 24680427 10.1016/j.ceb.2013.10.009 PMC3971390

[acer14396-bib-0024] King IFG , Eddison M , Kaun KR , Heberlein U (2014) EGFR and FGFR pathways have distinct roles in *Drosophila* mushroom body development and ethanol‐induced behavior. PLoS One 9:e87714. 24498174 10.1371/journal.pone.0087714 PMC3909204

[acer14396-bib-0025] Kong EC , Woo K , Li H , Lebestky T , Mayer N , Sniffen MR , Heberlein U , Bainton RJ , Hirsh J , Wolf FW (2010a) A pair of dopamine neurons target the D1‐like dopamine receptor DopR in the central complex to promote ethanol‐stimulated locomotion in *Drosophila* . PLoS One 5:e9954. 20376353 10.1371/journal.pone.0009954 PMC2848596

[acer14396-bib-0026] Kottler B , Faville R , Bridi JC , Hirth F (2019) Inverse control of turning behavior by dopamine D1 receptor signaling in columnar and ring neurons of the central complex in *Drosophila* . Curr Biol 29:567–577.e6. 30713106 10.1016/j.cub.2019.01.017 PMC6384123

[acer14396-bib-0027] Krashes MJ , Waddell S (2008) Rapid consolidation to a radish and protein synthesis‐dependent long‐term memory after single‐session appetitive olfactory conditioning in *Drosophila* . J Neurosci 28:3103–3113. 18354013 10.1523/JNEUROSCI.5333-07.2008 PMC2516741

[acer14396-bib-0028] Lannutti BJ , Schneider LE (2001) Gprk2 controls cAMP levels in *Drosophila* development. Dev Biol 233:174–185. 11319866 10.1006/dbio.2001.0219

[acer14396-bib-0029] Lefkowitz RJ , Shenoy SK (2005) Transduction of receptor signals by beta‐arrestins. Science 308:512–517. 15845844 10.1126/science.1109237

[acer14396-bib-0030] Levin LR , Han PL , Hwang PM , Feinstein PG , Davis RL , Reed RR (1992a) The *Drosophila* learning and memory gene rutabaga encodes a Ca2+/Calmodulin‐responsive adenylyl cyclase. Cell 68:479–489. 1739965 10.1016/0092-8674(92)90185-f

[acer14396-bib-0031] Li H , Tao Y , Ma Li , Liu X , Ma Lan (2013) β‐Arrestin‐2 inhibits preference for alcohol in mice and suppresses Akt signaling in the dorsal striatum. Neurosci Bull 29:531–540. 23839051 10.1007/s12264-013-1350-y PMC5561959

[acer14396-bib-0032] Liu S , Liu Q , Tabuchi M , Wu MN (2016) Sleep drive is encoded by neural plastic changes in a dedicated circuit. Cell 165:1347–1360. 27212237 10.1016/j.cell.2016.04.013 PMC4892967

[acer14396-bib-0033] Ly S , Pack AI , Naidoo N (2018) The neurobiological basis of sleep: insights from *Drosophila* . Neurosci Biobehav Rev 87:67–86. 29391183 10.1016/j.neubiorev.2018.01.015 PMC5845852

[acer14396-bib-0034] Managò F , Espinoza S , Salahpour A , Sotnikova TD , Caron MG , Premont RT , Gainetdinov RR (2012) The role of GRK6 in animal models of Parkinson’s disease and L‐DOPA treatment. Sci Rep 2:301. 22393477 10.1038/srep00301 PMC3293148

[acer14396-bib-0035] Maas JW , Vogt SK , Chan GC , Pineda VV , Storm DR , Muglia LJ (2005) Calciumstimulated adenylyl cyclases are critical modulators of neuronal ethanol sensitivity. J Neurosci 25(16):4118–4126. 15843614 10.1523/JNEUROSCI.4273-04.2005 PMC6724953

[acer14396-bib-0036] Moore CAC , Milano SK , Benovic JL (2007) Regulation of receptor trafficking by GRKs and arrestins. Annu Rev Physiol 69:451–482. 17037978 10.1146/annurev.physiol.69.022405.154712

[acer14396-bib-0037] Moore MS , DeZazzo J , Luk AY , Tully T , Singh CM , Heberlein U (1998) Ethanol intoxication in *Drosophila*: genetic and pharmacological evidence for regulation by the cAMP signaling pathway. Cell 93:997–1007. 9635429 10.1016/s0092-8674(00)81205-2

[acer14396-bib-0038] Morean ME , Corbin WR (2010) Subjective response to alcohol: a critical review of the literature. Alcohol Clin Exp Res 34:385–395. 20028359 10.1111/j.1530-0277.2009.01103.x

[acer14396-bib-0039] Ogden SK , Fei DL , Schilling NS , Ahmed YF , Hwa J , Robbins DJ (2008) G protein Galphai functions immediately downstream of Smoothened in Hedgehog signalling. Nature 456:967–970. 18987629 10.1038/nature07459 PMC2744466

[acer14396-bib-0040] Park SK , Sedore SA , Cronmiller C , Hirsh J (2000) Type II cAMP‐dependent protein kinase‐deficient *Drosophila* are viable but show developmental, circadian, and drug response phenotypes. J Biol Chem 275:20588–20596. 10781603 10.1074/jbc.M002460200

[acer14396-bib-0041] Peng J , Wagle M , Mueller T , Mathur P , Lockwood BL , Bretaud S , Guo S (2009) Ethanol‐modulated camouflage response screen in zebrafish uncovers a novel role for cAMP and extracellular signal‐regulated kinase signaling in behavioral sensitivity to ethanol. J Neurosci 29:8408–8418. 19571131 10.1523/JNEUROSCI.0714-09.2009 PMC2722107

[acer14396-bib-0042] Perkins LA , Holderbaum L , Tao R , Hu Y , Sopko R , McCall K , Yang‐Zhou D , Flockhart I , Binari R , Shim H‐S , Miller A , Housden A , Foos M , Randkelv S , Kelley C , Namgyal P , Villalta C , Liu L‐P , Jiang X , Huan‐Huan Q , Wang X , Fujiyama A , Toyoda A , Ayers K , Blum A , Czech B , Neumuller R , Yan D , Cavallaro A , Hibbard K , Hall D , Cooley L , Hannon GJ , Lehmann R , Parks A , Mohr SE , Ueda R , Kondo S , Ni J‐Q , Perrimon N (2015) The transgenic RNAi project at Harvard Medical School: resources and validation. Genetics 201:843–852. 26320097 10.1534/genetics.115.180208 PMC4649654

[acer14396-bib-0043] Ray LA , Mackillop J , Monti PM (2010) Subjective responses to alcohol consumption as endophenotypes: advancing behavioral genetics in etiological and treatment models of alcoholism. Subst Use Misuse 45:1742–1765. 20590398 10.3109/10826084.2010.482427 PMC4703313

[acer14396-bib-0044] Renn SC , Armstrong JD , Yang M , Wang Z , An X , Kaiser K , Taghert PH (1999) Genetic analysis of the *Drosophila* ellipsoid body neuropil: organization and development of the central complex. J Neurobiol 41:189–207. 10512977

[acer14396-bib-0045] Robie AA , Hirokawa J , Edwards AW , Umayam LA , Lee A , Phillips ML , Card GM , Korff W , Rubin GM , Simpson JH , Reiser MB , Branson K (2017) Mapping the neural substrates of behavior. Cell 170:393–406.e28. 28709004 10.1016/j.cell.2017.06.032

[acer14396-bib-0046] Robinson G , Most D , Ferguson LB , Mayfield J , Harris RA , Blednov YA (2014) Neuroimmune pathways in alcohol consumption: evidence from behavioral and genetic studies in rodents and humans. Int Rev Neurobiol 118:13–39. 25175860 10.1016/B978-0-12-801284-0.00002-6 PMC4264574

[acer14396-bib-0047] Rodan AR , Kiger JA , Heberlein U (2002) Functional dissection of neuroanatomical loci regulating ethanol sensitivity in *Drosophila* . J Neurosci 22:9490–9501. 12417673 10.1523/JNEUROSCI.22-21-09490.2002 PMC6758036

[acer14396-bib-0048] Rodan AR , Rothenfluh A (2010) The genetics of behavioral alcohol responses in *Drosophila* . Int Rev Neurobiol 91:25–51. 20813239 10.1016/S0074-7742(10)91002-7 PMC3531558

[acer14396-bib-0049] Roman G , He J , Davis RL (2000) kurtz, a novel nonvisual arrestin, is an essential neural gene in *Drosophila* . Genetics 155:1281–1295. 10880488 10.1093/genetics/155.3.1281 PMC1461172

[acer14396-bib-0050] Scaplen KM , Mei NJ , Bounds HA , Song SL , Azanchi R , Kaun KR (2019) Automated real‐time quantification of group locomotor activity in *Drosophila* melanogaster. Sci Rep 9 10.1038/s41598-019-40952-5. PMC6418093 30872709

[acer14396-bib-0051] Schneider LE , Spradling AC (1997) The *Drosophila* G‐protein‐coupled receptor kinase homologue Gprk2 is required for egg morphogenesis. Development 124:2591–2602. 9217001 10.1242/dev.124.13.2591

[acer14396-bib-0052] Scholz H (2005) Influence of the biogenic amine tyramine on ethanol‐induced behaviors in *Drosophila* . J Neurobiol 63:199–214. 15729684 10.1002/neu.20127

[acer14396-bib-0053] Scholz H , Franz M , Heberlein U (2005) The hangover gene defines a stress pathway required for ethanol tolerance development. Nature 436:845–847. 16094367 10.1038/nature03864 PMC1364536

[acer14396-bib-0054] Scholz H , Ramond J , Singh CM , Heberlein U (2000) Functional ethanol tolerance in *Drosophila* . Neuron 28:261–271. 11086999 10.1016/s0896-6273(00)00101-x

[acer14396-bib-0055] Schuckit MA (1994) Alcohol sensitivity and dependence. EXS 71:341–348. 8032165 10.1007/978-3-0348-7330-7_34

[acer14396-bib-0057] Shaw PJ , Cirelli C , Greenspan RJ , Tononi G (2000) Correlates of sleep and waking in *Drosophila* melanogaster. Science 287:1834–1837. 10710313 10.1126/science.287.5459.1834

[acer14396-bib-0058] Tanoue S , Krishnan P , Chatterjee A , Hardin PE (2008) G protein‐coupled receptor kinase 2 is required for rhythmic olfactory responses in *Drosophila* . Curr Biol 18:787–794. 18499458 10.1016/j.cub.2008.04.062 PMC2474769

[acer14396-bib-0059] Tipping M , Kim Y , Kyriakakis P , Tong M , Shvartsman SY , Veraksa A (2010) β‐arrestin Kurtz inhibits MAPK and Toll signalling in *Drosophila* development. EMBO J 29:3222–3235. 20802461 10.1038/emboj.2010.202 PMC2957207

[acer14396-bib-0060] Topalidou I , Cooper K , Pereira L , Ailion M (2017) Dopamine negatively modulates the NCA ion channels in *C*.* elegans* . PLoS Genet 13:e1007032. 28968387 10.1371/journal.pgen.1007032 PMC5638609

[acer14396-bib-0061] Troutwine BR , Ghezzi A , Pietrzykowski AZ , Atkinson NS (2016) Alcohol resistance in *Drosophila* is modulated by the Toll innate immune pathway. Genes Brain Behav 15:382–394. 26916032 10.1111/gbb.12288 PMC4991213

[acer14396-bib-0062] Urizar NL , Yang Z , Edenberg HJ , Davis RL (2007) *Drosophila* homer is required in a small set of neurons including the ellipsoid body for normal ethanol sensitivity and tolerance. J Neurosci 27:4541–4551. 17460067 10.1523/JNEUROSCI.0305-07.2007 PMC6672997

[acer14396-bib-0063] Valanne S , Myllymäki H , Kallio J , Schmid MR , Kleino A , Murumägi A , Airaksinen L , Kotipelto T , Kaustio M , Ulvila J , Esfahani SS , Engström Y , Silvennoinen O , Hultmark D , Parikka M , Rämet M (2010) Genome‐wide RNA interference in *Drosophila* cells identifies G protein‐coupled receptor kinase 2 as a conserved regulator of NF‐kappaB signaling. J Immunol 1950(184):6188–6198. 10.4049/jimmunol.1000261 20421637

[acer14396-bib-0064] van der Linde K , Fumagalli E , Roman G , Lyons LC (2014) The FlyBar: administering alcohol to flies. J Vis Exp 18:50442. 10.3791/50442 PMC4193141 24895004

[acer14396-bib-0065] Wand G , Levine M , Zweifel L , Schwindinger W , Abel T (2001) The cAMP‐protein kinase A signal transduction pathway modulates ethanol consumption and sedative effects of ethanol. J Neurosci 21:5297–5303. 11438605 10.1523/JNEUROSCI.21-14-05297.2001 PMC6762861

[acer14396-bib-0066] Wolf FW , Rodan AR , Tsai LT‐Y , Heberlein U (2002) High‐resolution analysis of ethanol‐induced locomotor stimulation in *Drosophila* . J Neurosci 22:11035–11044. 12486199 10.1523/JNEUROSCI.22-24-11035.2002 PMC6758417

[acer14396-bib-0067] Wu JS , Luo L (2006) A protocol for dissecting *Drosophila* melanogaster brains for live imaging or immunostaining. Nat Protoc 1:2110–2115. 17487202 10.1038/nprot.2006.336

[acer14396-bib-0068] Xu S , Chan T , Shah V , Zhang S , Pletcher SD , Roman G (2012) The propensity for consuming ethanol in *Drosophila* requires rutabaga adenylyl cyclase expression within mushroom body neurons. Genes Brain Behav 11:727–739. 22624869 10.1111/j.1601-183X.2012.00810.x PMC3404234

[acer14396-bib-0069] Yang X , Oswald L , Wand G (2003) The cyclic AMP/protein kinase A signal transduction pathway modulates tolerance to sedative and hypothermic effects of ethanol. Alcohol Clin Exp Res 27:1220–1225. 12966313 10.1097/01.ALC.0000081626.02910.19

[acer14396-bib-0070] Zhang J , Ferguson SS , Barak LS , Bodduluri SR , Laporte SA , Law PY , Caron MG (1998) Role for G protein‐coupled receptor kinase in agonist‐specific regulation of mu‐opioid receptor responsiveness. Proc Natl Acad Sci USA 95:7157–7162. 9618555 10.1073/pnas.95.12.7157 PMC22772

